# A Variational Stacked Autoencoder with Harmony Search Optimizer for Valve Train Fault Diagnosis of Diesel Engine †

**DOI:** 10.3390/s20010223

**Published:** 2019-12-31

**Authors:** Kun Chen, Zhiwei Mao, Haipeng Zhao, Zhinong Jiang, Jinjie Zhang

**Affiliations:** 1 Key Lab of Engine Health Monitoring-Control and Networking of Ministry of Education, Beijing University of Chemical Technology, Beijing 100029, China; chenkun_chn@163.com (K.C.); 2017400141@mail.buct.edu.cn (H.Z.); 2 Beijing Key Laboratory of High-End Mechanical Equipment Health Monitoring and Self-Recovery, Beijing University of Chemical Technology, Beijing 100029, China; jiangzn@mail.buct.edu.cn (Z.J.); zhangjinjie@mail.buct.edu.cn (J.Z.)

**Keywords:** deep learning, autoencoder, harmony search optimizer, diesel engine, fault diagnosis

## Abstract

Diesel engine fault diagnosis is vital due to enhanced reliability and economic efficiency requirements. The extracted features in traditional fault diagnosis are constructed manually, which is very cumbersome because of the requirement for lots of expertise. To handle this issue, this paper proposed a variational stacked autoencoder (VSAE) to adaptively extract features from angular domain signals. As an unsupervised algorithm, VSAE can extract high-level features with the help of multiple encoding layers. Layer-wise pre-training and fine-tuning are introduced to get a better network initialization value. Moreover, the dropout technique and the batch normalization technique are carried out to prevent over-fitting and implement fast convergence. Finally, the harmony search optimizer (HSO) algorithm is introduced to get an appropriate hyper-parameter setting in the VSAE model, as well as make adaptive adjustment of the network structure. In order to verify the proposed method, the valve train fault data is collected on the diesel engine test rig under twelve operating conditions. The results indicate that the proposed scheme can effectively diagnose different degrees of intake valve fault, exhaust valve fault, and coupling fault under various operating conditions. Furthermore, the classification accuracy improved from 94.10% to 98.85%VSAE compared with stacked autoencoder (SAE) and some other traditional fault diagnosis algorithms.

## 1. Introduction

As a critical power source, diesel engines are an irreplaceable part of heavy industry, agriculture, nuclear power, and other fields. It is common that diesel engines are subject to fault because of their complex internal structure and harsh operating environments [1,2,3]. This may lead to failure of the entire system if the fault is not diagnosed in time, threatening the operator’s safety and causing great economic loss. For example, the valve train is a particularly important part of the diesel engine, which is mainly composed of intake valve, exhaust valve, rocker arm, pushrod, and tappet. A healthy valve train guarantees correct timing of the valve, that is, intake and exhaust at the time of default. Besides, a normal valve train clearance is also vital for thermal compensation. However, because of the impact of various vibrations sources, the valve clearance frequently trends to increase due to component wear after too many operating hours, reducing the efficiency of the diesel engine. It can even deteriorate into a valve fracture fault, reducing reliability [4,5,6,7]. Therefore, research on diesel engine health monitoring and fault diagnosis is valuable and urgently needed. Fortunately, related research work is extensively investigated and many algorithms are developed to diagnose fault based on thermal parameters, vibration signals, acoustic emission signals, or some other signals [8,9,10].

The basic scheme of fault diagnosis generally consists of three successive stages: data acquisition, feature extraction, and decision making. Feature extraction, which is manually performed with the help of signal processing methods and statistic methods, plays a critical role in the performance of the diagnosis. It is a very common technical route to perform time domain, frequency domain, and time-frequency domain analysis based on vibration signals obtained by installing acceleration sensors. There are many existing methods based on the above, such as Fourier transform (FT), short-time Fourier transform (STFT), continuous wavelet transform (CWT), Wigner Ville distribution (WVD), Choi–Williams distribution (CWD), singular value decomposition (SVD), empirical mode decomposition (EMD), variational mode decomposition (VMD), and sparse time-frequency analysis (STFA). In [11], STFT, WVD, and CWD were utilized, respectively, for detailed scrutiny of vibrations generated by an under-load engine, and the root mean square (RMS) and kurtosis of vibration signals were calculated to perform the fault diagnosis of injectors. In [12], discriminative non-negative matrix factorization (DNMF) was carried out to capture the features of time-frequency image, which were then passed to k-nearest neighbors (KNN) to implement the diagnosis of the diesel valve fault. In [13], STFT and CWT were developed to mine the features of the time domain signals, and the maximum, mean, and energy of the engine vibration were found increased significantly in the frequency band of 3–4.7 kHz when the piston has a scratching fault. In [14], an improved VMD was proposed for signal decomposition, and then instantaneous energy distribution-permutation entropy was implemented for feature extraction. Unfortunately, in spite of many existing algorithms on feature extraction, these processing and feature extraction algorithms are difficult to absolutely adapt to specific signals. CWT has achieved satisfactory results in the time-frequency analysis of non-stationary signals, but the wavelet basis and decomposition scale need to be manually selected, which restricts the performance of self-adaptation. EMD can adaptively decompose signals into several intrinsic mode functions according to their characteristics. Nevertheless, it has the flaw of the mode mixing and the end effect. The number of modes in VMD plays a critical role in the decomposition result, but the parameter is selected based on prior-knowledge. In general, signal processing based on expertise or prior-knowledge will inevitably lead to information missing. The experience limitation of the fault mechanism can make the performance of these algorithms unsatisfactory.

Fortunately, with the deepening application of machine learning in the industrial field, some emerging intelligent fault diagnosis algorithms have demonstrated inspiring results [15]. Among them, the emergence of the stacked autoencoder (SAE) has brought us a new method of feature extraction, that is, the hierarchical extraction of deep abstract features directly from the raw signal via unsupervised or semi-supervised training [16]. Compared to traditional manually chosen features, the features extracted by SAE adaptively retain the most representative information based on the characteristics of a signal. In addition, a lot of signal processing work is simplified due to the end-to-end network structure. Therefore, SAE has been employed by many scholars to address machinery fault diagnosis, and the performance is remarkable. In [17], the features of fault bearing under different crack sizes were obtained with the help of SAE, and not surprisingly, the fault diagnosis of bearing crack was effectively performed. In [18], a SAE feature mining method was developed to diagnose gearbox fault from frequency-domain signals, and two gearbox datasets were collected to confirm the effectiveness of the proposed method. In [19], a stacked denoising autoender (SDAE) was established to carry out layer-wise feature extraction from vibration signals in the frequency domain. Then, principal component analysis was used to verify the feature extraction ability. In [20], a SDAE was introduced to automatically learn in-depth features from complex data. Combined with an improved regularization method, the model achieved an impressive result. Moreover, the regularization parameters changed depending on the layers of the SDAE. However, most of the above studies are the introduction and application of SAE, or the improvement of the structure in SAE, or the optimization of specific hyper-parameters. Because of the deep network structure of SAE, there are many hyper-parameters that can have a significant influence on the performance of fault diagnosis. The multi-parameter joint optimization in the SAE model is still an urgent task for current research. Besides, when SAE is utilized for reciprocating mechanical feature extraction, the test data that is quite different from the training data in the operating condition may lead to an insufficient performance. Therefore, the new network design of SAE still should be investigated to adjust to multiple operating conditions.

In this paper, a variational stacked autoencoder with harmony search optimizer (HSO–VSAE) method is carried out to handle the diagnosis of a diesel engine under multiple operating conditions. The proposed algorithm is verified by the case of a 12-cylinder diesel engine data that consists of a normal intake valve train fault and an exhaust valve train fault under 12 operating conditions. The main contributions of this paper can be summed up as follows: (1) In order to overcome the dependence of prior knowledge in traditional feature extraction, a novel variational stacked autoencoder (VSAE) model is proposed to mine high-level features adaptively from angular domain signals considering multiple operating conditions. (2) The dropout technique and the batch normalization technique are introduced to get over the flaw of over-fitting and the internal covariate shift problem in the deep layers of SAE. (3) In order to achieve the multi-parameter joint optimization in the proposed model, the harmony search optimizer (HSO) algorithm is adopted to adjust the network structure for a good match with the given data set.

The rest organization of the paper is as follows. In Section 2, the theoretical background of VSAE and HSO is introduced. Section 3 presents the proposed HSO–VSAE method for the fault diagnosis of diesel engine. Section 4 describes the diesel engine test rig and the data obtained. Section 5 details the experimental results of valve train diagnosis based the proposed method. The conclusions are presented in Section 6.

## 2. Theoretical Background

### 2.1. Variational Stacked Autoencoder

#### 2.1.1. Autoencoder

**Figure 1:**
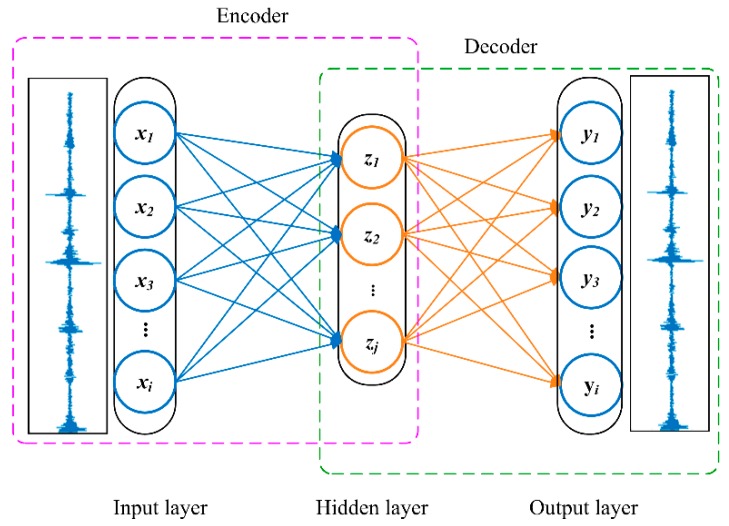
The basic architecture of an autoencoder (AE).

Autoencoder (AE) is a type of artificial neural network that is symmetric in terms of network structure. It is a very effective feature extraction method based on unsupervised learning. AE is composed of encoder and decoder, as shown in Figure 1. The encoder compresses the input data by nonlinear mapping, and the decoder reconstructs the input from the extracted features. After encoding and decoding, the output layer attempts to learn a set of vectors that are as close as possible but not identical to the input vector. At this point, the outputs of the hidden layer are the features that AE automatically extracts through unsupervised learning.

The encoding process can be described by Equation (1)

(1) $$Z=\sigma_{1}\left(W_{1}\cdot X+b_{1}\right)$$

where *X* and *Z* are the vectors of the input layer and the hidden layer, respectively. *W*_1_ and *b*_1_ are the weight matrix and basis vector, respectively. *σ*_1_ is the activation function of encoder.

The decoding process can be described by Equation (2)

(2) $$Y=\sigma_{2}\left(W_{2}\cdot Z+b_{2}\right)$$

where *Y*, *W*_2_, and *b*_2_ are the output vector, weight matrix, and basis vector, respectively. *σ*_2_ is the activation function of decoder.

#### 2.1.2. Variational Autoencoder

**Figure 2:**
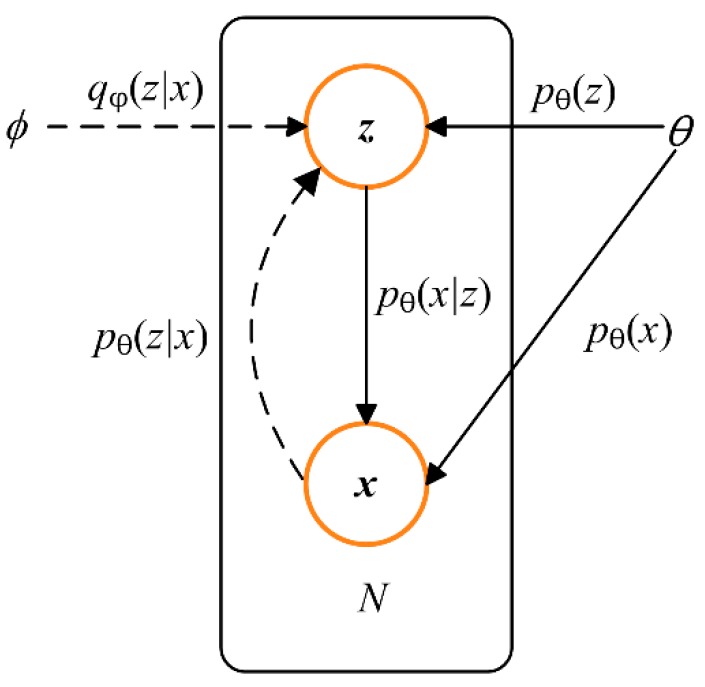
The probability graph model of variational autoencoder (VAE).

Variational autoencoder (VAE) has been widely applied in process monitoring and fault diagnosis [21,22,23] since it was firstly proposed in [24]. As shown in Figure 2, VAE generates *x* by sampling from the distribution of latent variable *z*. In Figure 2, *p_θ_*(*z*) is the prior probability of the latent variable *z*, which denotes the original distribution of *z*; *p_θ_*(*x*) is the distribution of the data set *x* that needs to be generated; and *p_θ_*(*z*|*x*) represents the posterior distribution of *z*, which is also called encoder. For the reason that *p_θ_*(*z*|*x*) is intractable to solve in practice, the variational approximation *q_φ_*(*z*|*x*) is often used to replace it.

In VAE, the *q_φ_*(*z*|*x*) is usually a Gaussian distribution, and the KL divergence between *p_θ_*(*z*|*x*) and *q_φ_*(*z*|*x*) can be described by Equation (3)

(3) $$D_{KL}[q_{\varphi }(z|x)∥p_{\theta }(z|x)]=E_{q_{\varphi }(z|x)}[\log q_{\varphi }(z|x)-\log p_{\theta }(z|x)]$$

According to the Bayes rule, Equation (3) can be written as

(4) $$\begin{array}{ll} D_{KL}[q_{\varphi }(z|x)∥p_{\theta }(z|x)]= & E_{q_{\varphi }(z|x)}[\log q_{\varphi }(z|x)-\log p_{\theta }(x|z)-\log p_{\theta }(z)]\\ & +\log p_{\theta }(x) \end{array}$$

Since the KL divergence is non-negative, the formula can be written as

(5) $$\log p_{\theta }(x)\geq L(\theta ,\varphi ;x)$$

where

(6) $$\begin{array}{cc} L(\theta ,\varphi ;x) & =-E_{q_{\varphi }(z|x)}[\log q_{\varphi }(z|x)-\log p_{\theta }(x|z)-\log p_{\theta }(z)]\\ & =-D_{KL}[q_{\varphi }(z|x)∥p_{\theta }(z)]+E_{q_{\varphi }(z|x)}[\log p_{\theta }(x|z)] \end{array}$$

In Equation (6), L(θ,φ;x) is called the variational lower bound of $\log p_{\theta }(x)$, which is also the loss function of VAE. The first term is the regularization term. The second term denotes the reconstruction error.

By assuming that $p_{\theta }(z)∼N(0,I)$, $q_{\varphi }(z|x)∼N(\mu ,\sigma^{2})$, the first term on the right hand side of Equation (6) can be calculated as

(7) $$-D_{KL}[q_{\varphi }(z|x)∥p_{\theta }(z)]=\frac{1}{2}\sum_{j=1}^{d}(1+\log{\left(\sigma_{j}\right)}^{2}-{\left(\mu_{j}\right)}^{2}-{\left(\sigma_{j}\right)}^{2})$$

where *d* is the dimensionality of the distribution.

The second term on the right hand side of Equation (6) can be calculated by Equation (8)

(8) $$E_{q_{\varphi }(z|x)}[\log p_{\theta }(x|z)]=\frac{1}{L}\sum_{l=1}^{L}\log p_{\theta }(x|z^{\left(l\right)})$$

where *L* is chosen to be 1 in this paper, and

(9) $$\left\{ \begin{array}{l} z^{\left(l\right)}=\mu +\sigma ⊙\varepsilon^{\left(l\right)}\\ \varepsilon^{\left(l\right)}∼N(0,I) \end{array}\right.$$

Thus, the VAE loss can be obtained by Equation (6), and even if the sampling method is taken in the process of solving the loss, the back propagation can also be applied in the neural network as usual.

#### 2.1.3. VSAE Model

In order to achieve better high-level feature extraction, multiple AEs are usually stacked to build a SAE model. Compared to AE, the SAE has more complex network structure and better nonlinear fitting ability, which allow it to perform feature extraction directly from the raw data when it is utilized in fault diagnosis based on vibration signals. However, SAE only extracts features by nonlinear transformation, and it is difficult for SAE to model the whole latent feature space. Fortunately, this problem can be solved by introducing VAE. As a generation model, VAE can complete the modeling of input data by characterizing the distribution of features rather than the transformations used in extract features.

**Figure 3:**
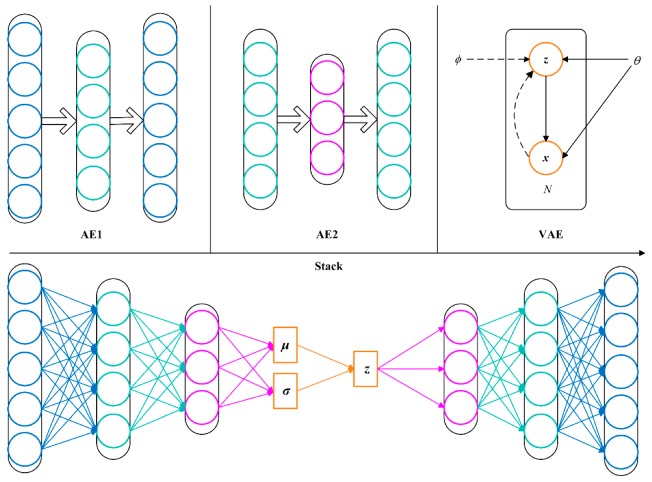
The construction process of variational stacked autoencoder (VSAE) model.

VSAE is a novel neural network by embedding VAE in SAE, which combines the merits of these two models. As shown in Figure 3, two AEs are pre-trained in succession, and then AE1, AE2, and VAE are stacked to form the VSAE model. Finally, fine-tuning of parameters is performed. In the VSAE, the features of input signals are extracted hierarchically by AE1 and AE2. Then, the distribution of latent feature space is modeled by a VAE network. Specifically, the standard deviation vector ***σ*** and mean vector ***μ*** of several Gaussian distributions are obtained with the help of two encoders, then samplings are performed from a standard Gaussian distribution N(0,I), and the latent feature vector ***z*** is constructed by Equation (9), which indicates the process of reparameterization technique. Finally, after the decoders formed by multiple fully connected layers, the features are reconstructed into signals that are as equal as possible to the input signals. This VSAE model extracts the most essential features of the signal. In terms of mechanical vibration signals, satisfactory performance can be achieved even under varying operating conditions.

### 2.2. Harmony Search Optimizer

HSO algorithm is an emerging intelligence optimization algorithm, which is inspired by the principle of the band rehearsal [25,26]. In the rehearsal of the band, the pitch of each instrument is repetitively adjusted to form a beautiful harmony. HSO algorithm is simple and easy to implement, and requires fewer adjustment parameters. Therefore, the HSO algorithm has been widely used in the fields of combinatorial optimization problems since it was proposed. The procedures of HSO algorithm are as follows:

- **Step 1:** Define the objective function *f*(*X*), and initialize the harmony memory size *HMS*, the harmony memory considering rate *HMCR*, the pitch adjusting rate *PCR*, fine-tuning bandwidth *B*, and the maximum number of iterations *MAX*;
- **Step 2:** Determine the solution space, and randomly generate *HMS* group parameters from the solution space to form a harmony memory;
- **Step 3:** Randomly generate a variable *R*_1_ from [0, 1]. If *R*_1_ > HMCR, a new harmony is randomly selected from the solution space; otherwise, a new harmony is randomly selected from the harmony memory.
- **Step 4:** If the new harmony is generated in the harmony memory, a variable *R*_2_ is randomly generated from [0, 1] and compared with the *PCR*. If *R*_1_ > *PCR*, the harmony is not adjusted; otherwise, the harmony is fine-tuned by
  (10) $$X_{new}=X_{new}\pm R_{3}\times B$$
  where *X_new_* is the adjusted harmony. *R*_3_ is a random number between [0, 1], and *B* is the adjustment bandwidth which is set to 2 in this paper.
- **Step 5:** If the resulting new harmony is better than the worst solution in the harmony memory, replace the worst harmony with the new harmony and update the harmony memory.
- **Step 6:** Repeat the above process until the number of iterations is *MAX*.

**Figure 4:**
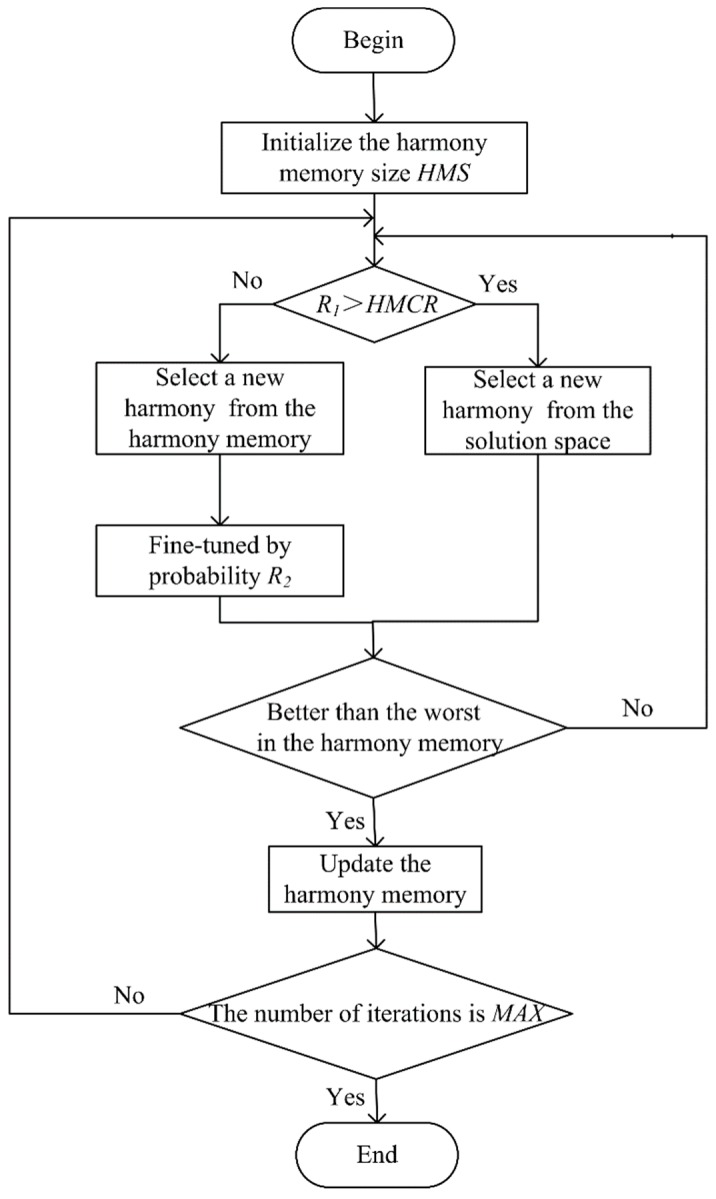
The flow chart of harmony search optimizer (HSO) algorithm.

The flow chart of HSO algorithm is shown in Figure 4.

## 3. The Proposed HSO–VSAE Method

**Figure 5:**
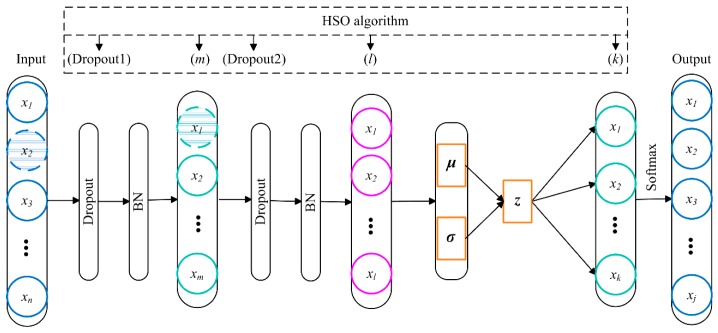
The proposed variational stacked autoencoder with harmony search optimizer (HSO–VSAE) scheme.

The deep network structure of VSAE may introduce some flaws while improving the performance of feature extraction, such as the over-fitting problem and the “internal covariate shift” problem. Therefore, the dropout technique [27] and the batch normalization (BN) technique are introduced in this study to improve the generalization ability and convergence rate of the VSAE model. With the help of these techniques, the VSAE can converge quickly after fine-tuning, extract features hierarchically, and classify faults. However, there is still a concern that needs to be emphasized: some hyper-parameters in this model have a significant influence on the feature extraction effect, and inappropriate parameter values will degrade the performance of the VSAE model. On the other hand, fixed hyper-parameter settings are obviously not reasonable enough for different data sets. By introducing the HSO algorithm into the VSAE, the network structure can be adaptively adjusted according to different data sets, further improving the high-level feature extraction performance of the VSAE. The HSO–VSAE fault diagnosis model is shown in Figure 5.

In this HSO–VSAE fault diagnosis frame, a VSAE model is built first as in Figure 3. Then, the decoder part of the VSAE is discarded when the pre-training is completed, and a fully connected layer and a softmax layer are sequentially added to classify the faults. After adding the dropout layers—the BN layers—fine-tuning is performed to complete the training of the VSAE model. For the node drop ratios of the first two layers and the number of hidden layer nodes, a total of five important hyper-parameters in the VSAE, namely, *Dropout1*, *Dropout2*, *m*, *l*, and *k*. The initial range of values is determined based on the experiment or existing knowledge. Then, the solution space and harmony memory are established according to the range of each parameter. Finally, five hyper-parameters are adaptively chosen by the HSO algorithm in accordance with the distribution characteristics of data set. Therefore, the proposed HSO–VSAE method can achieve both satisfactory fault diagnosis performance and generalization capability even under varying operating conditions.

## 4. Test Rig and Data Description

**Figure 6:**
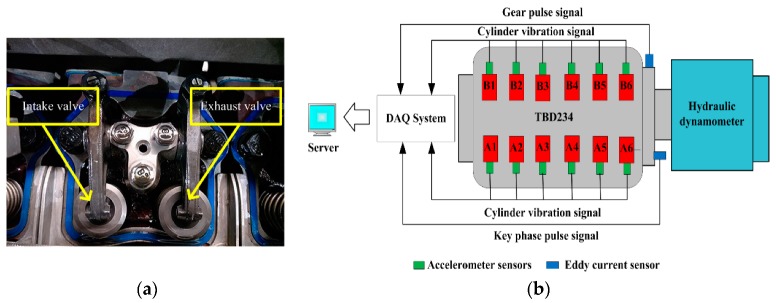
Valve train and the main test rig components: (**a**) intake valve and exhaust valve and (**b**) the sketch of diesel engine test rig and sensors positioning.

The TBD234 test rig is a 12-cylinder piston diesel engine designed by Henan Diesel Engine Industry Co. Ltd., (Luoyang, China). The key parameters of the diesel engine are shown in Table 1. A BH5011 acceleration sensor, designed by Beijing Bohua Technology Co. Ltd., (Beijing, China), is installed in the vertical direction of the cylinder head of each cylinder to obtain a cylinder vibration signal. To obtain an instantaneous speed signal and a key phase signal, a Bently 3300 XL eddy current sensor is respectively installed in the radial direction and the axial direction of the flywheel, which is directly connected to the crankshaft. Test data is collected by the BH5000E monitoring system designed by Beijing Bohua Technology Co. Ltd., (Beijing, China). The sampling is performed in the time domain at a sampling frequency of 51.2 kHz. The main components of the test rig are shown in Figure 6.

As a kind of motion mechanism, the valve train of the diesel engine often has an abnormal increase in the valve clearance due to wear, resulting in economic loss. Thence, the abnormality of the intake and exhaust valve clearance is the target fault of this experiment. In the healthy state, the normal intake valve clearance and exhaust valve clearance should be 0.3 mm and 0.5 mm, respectively. In this experiment, the clearances are adjusted by the feeler gauge to simulate different degrees of valve fault. The experiment simulates seven states of valve clearance, that is, normal state (NS), serious intake valve fault (SIF), serious exhaust valve fault (SEF), serious intake and exhaust valves fault (SIE), minor intake valve fault (MIF), minor exhaust valve fault (MEF), and minor intake and exhaust valves fault (MIE). The specific fault valve train clearance setting is summarized in Table 2. In this experiment, the vibration data of 12 operating conditions are collected along with 80 data files for each operating condition. Therefore, 960 data files are obtained for each kind of fault. The detailed operating conditions are shown in Table 3.

**Figure 7:**
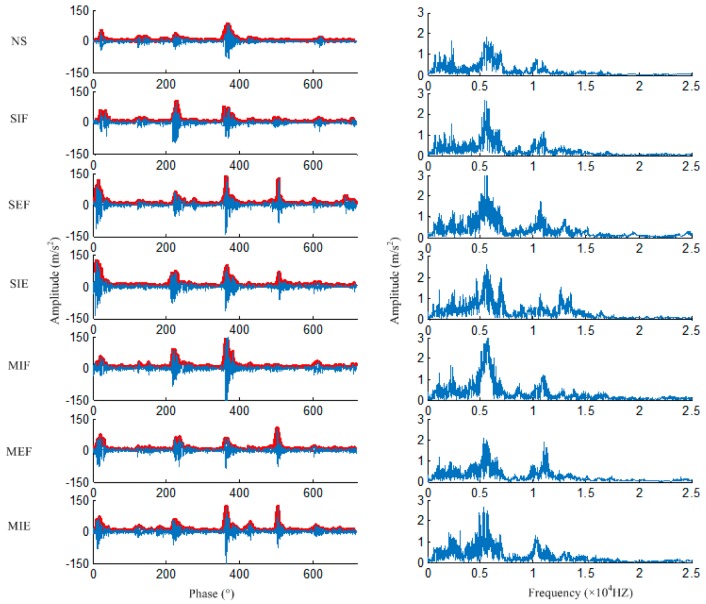
Angular domain, frequency domain signals, and the envelope.

For the TBD234 diesel engine, one operating cycle consists of 720 degrees in angular domain no matter how the conditions change. For time domain signal, the length of each data file in each cycle is different under different operating conditions, which does not meet the needs of the model input. Therefore, the instantaneous speeds of engine are first obtained with the help of an eddy current sensor; then, the angular domain signals are obtained by resampling the time domain signal to the phase of 0~720° by linear interpolation. For each operating condition, the length of each data file is fixed at 3600. The envelopes are extracted in the time domain by the second order extremum method and then also resampled to the angular domain, as shown by the red lines in Figure 7. The frequency domain signals are obtained by a Fourier transform of the time domain signal. Figure 7 shows the angular domain signals, the frequency domain signals, and the envelopes in seven fault categories at 1500 rpm and 1300 N·m.

## 5. Experimental Verification

### 5.1. Control Experiments

#### 5.1.1. Model Input

**Figure 8:**
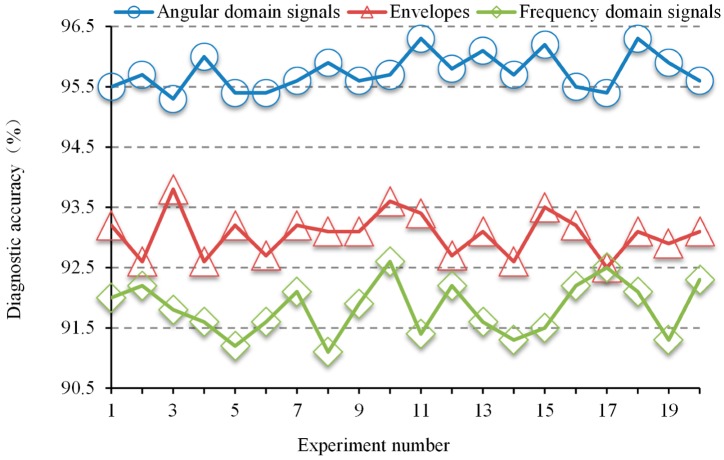
The diagnostic accuracy under three input modes.

To study the effect of different input methods on classification accuracy, three groups of control experiments were set up. The first group took the angular domain signals as input, the second group took the frequency domain signals as input, and the third group took the envelopes as input. In these groups, 25% of the data set was used for the pre-training of VSAE model, another 25% was used for fine-tuning, and the rest 50% of the data was used for testing. The data set contained all 12 operating conditions. After adding the softmax layer, the network structure was 3600-512-256-20-10-64-7. In the pre-training, the iteration times of AE1, AE2, and VAE were 200, 100, and 50, respectively, the node discarding ratios *Dropout1* and *Dropout2* were both set to 0.1, and the number of iterations of fine-tuning SAE was 20 steps. In order to consider the influence of randomness, each group of experiments was repeated 20 times, and the diagnostic accuracy is shown in Figure 8. It can be seen from Figure 8 that when the angular domain signals were selected as the input, the diagnostic accuracy was 95.3~96.3%. When the frequency domain signals were selected as the input, the diagnostic accuracy was 91.1~92.5%. When the envelopes were selected as the input, the diagnostic accuracy was 92.5~93.8%. The results indicate that better diagnostic performance can be achieved with the angular domain signals as input. Representative input is very helpful for the feature extraction of each hidden layer. The larger the proportion of useful information in the input, the easier it is to retain the most effective principal component when extracting features hierarchically. Although the envelope can attenuate the effects of background noise in the vibration signals compared to the angular domain signals, the extraction of the envelope also leads to a loss of some key feature information, which is useful for fault diagnosis, such as frequency domain and time-frequency domain information. In the same way, the use of spectrum as input also results in the omission of information. The experimental results show that due to the limited cognition of the fault mechanism, pre-processing based on expert experience causes a certain degree of information loss to the input signals. In contrast, VSAE has obvious diagnostic advantages because it retains most information of the angular domain signals.

#### 5.1.2. Dropout Rate

**Figure 9:**
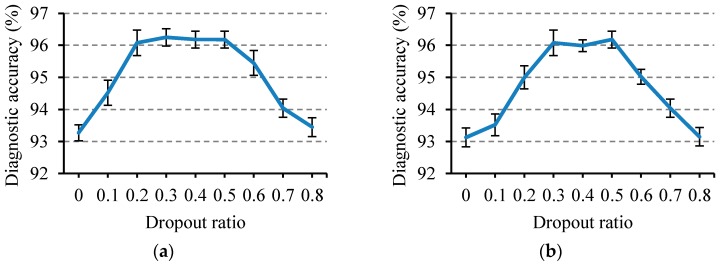
Diagnostic accuracy when the dropout parameters of the first two layers change: (**a**) the dropout ratio of the first layer *Dropout1* and (**b**) the dropout ratio of the second layer *Dropout2*.

The selection of dropout parameters has an effect on the performance of the VSAE model. It is difficult to produce a good regularization effect, and the model performance improvement is not obvious when the parameter is too small. However, when the ratio is too large, it introduces too much noise, which may also make the model not perform well. In order to study the effect of the dropout parameters on the VSAE fault diagnosis results, a group of control experiments was set up, as shown in Figure 9.

In the control experiments, the dropout parameters were raised from 0 to 0.8 with a gradient of 0.1, and the remaining parameters remained unchanged. Twenty sets of repeated experiments were performed under each parameter setting, and the average diagnostic accuracy and 95% confidence interval were calculated. It can be clearly seen from the Figure 9 that the diagnostic accuracy increases first and then decreases with the increase of the dropout parameter, and the best diagnostic effect can be obtained when the dropout ratio is between 0.2 and 0.5.

### 5.2. Parameter Optimization Based on HSO Algorithm

The number of nodes in the hidden layer in the VSAE network is also critical for the extraction of high-level features. However, there is currently no general rule for the determination of value. According to the existing experience of value [28], the number of hidden layer nodes should be reduced layer by layer. It is easier to get better results when the number of nodes is reduced by half or more. In addition, when there are multiple hyper-parameters in the VSAE model, it is unreasonable to analyze the optimal value of a single hyper-parameter in isolation by the method of control experiment. Combinatorial optimization methods need to be employed to optimize multiple hyper-parameters simultaneously. According to the existing experience mentioned above, the number of nodes—*m*, *l*, and *k*—of each hidden layer were preliminarily set to 400~800, 200~400, and 50~100, respectively. *Dropout1* and *Dropout2* were preliminarily set to 0.2~0.5, and the solution space was established according to the range of the five hyper-parameters. *HMS* = 25, *HMCR* = 0.75, and *PCR* = 0.5 were initialized, with diagnostic accuracy as the objective function of HSO optimization and the maximum number of iterations set at 100. After the optimization of the HSO algorithm, the values of the partial hyper-parameters in the harmony memory are shown in Table 4.

As can be seen from Table 4, for the experimental data set, when *Dropout1*, *Dropout2*, *m*, *l*, and *k* are in different combinations, the accuracy obtained is different. When these parameters are optimized by the HSO algorithm, the efficiency of the VSAE model is significantly improved compared to the previous results in Section 5.1. When the data set changes, the hyper-parameters in the HSO–VSAE model will also change. The introduction of the HSO algorithm can make the network structure adaptively adjust according to the characteristics of the case data, and select proper parameter combination to achieve optimal fault diagnosis performance.

### 5.3. Feature Visualization

**Figure 10:**
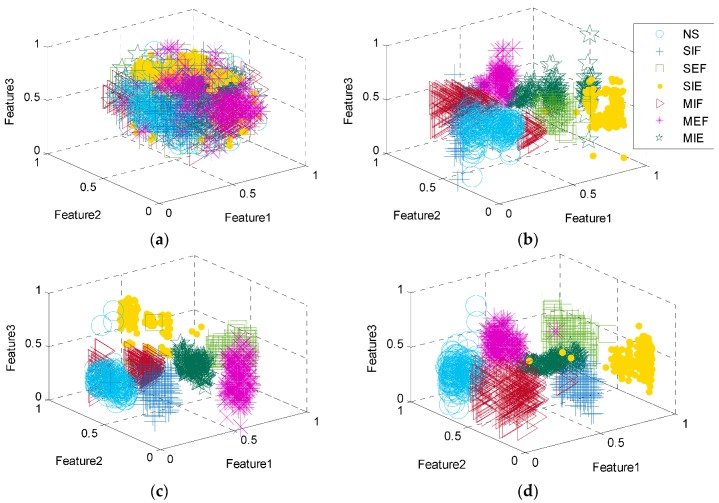

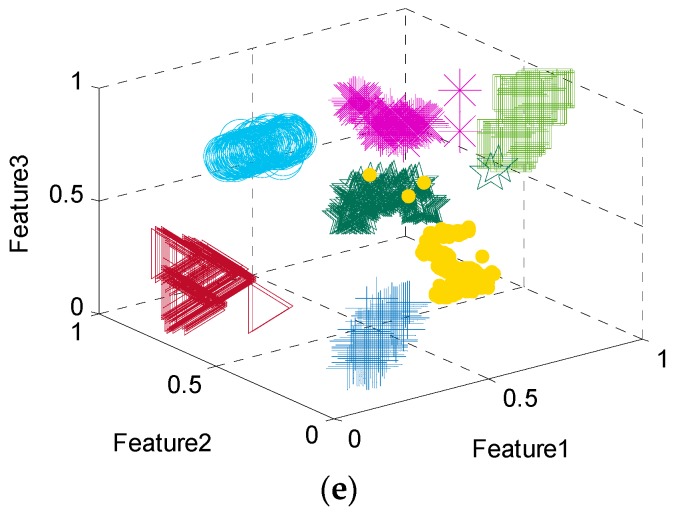
Features of each layer extracted by T-Distribution Stochastic Neighbor Embedding (t-SNE): (**a**) the input layer, (**b**) the second layer, (**c**) the third layer, (**d**) the latent feature space ***z***, and (**e**) the last hidden layer.

To confirm the ability of HSO–VSAE in extracting features hierarchically, and to analyze the variation of feature distribution of each layer, the results of T-Distribution Stochastic Neighbor Embedding (t-SNE) visualization technology [29] are shown in Figure 10. It can be clearly seen that the clustering capability of each category will gradually enhance after each layer of the HSO–VSAE, indicating that the proposed method has a superior deep feature extraction performance.

As can be seen in Figure 10, the misidentification of different categories is very serious in the input layer, and it is impossible to distinguish any category by t-SNE visualization technology. The same category of data files begin to aggregate after the second layer, but the separation performance is still unsatisfactory. The separation between different categories becomes more obvious after the third layer, but the aggregation of same category is unsatisfactory. For example, the MIF is clustered into two locations and the SIE is somewhat scattered. After the latent space is constructed, the distributions of different categories of data files are well distinguished. After the last hidden layer, the data files are completely separated. The distance between the different categories is significant, and the aggregation of the same category is profound. In addition, the HSO–VSAE can still work effectively under twelve operating conditions, indicating that the deep features extracted hierarchically eliminate the influence of changing operating conditions.

### 5.4. Analysis and Discussion of Diagnostic Results

#### 5.4.1. Detailed Analysis

**Figure 11:**
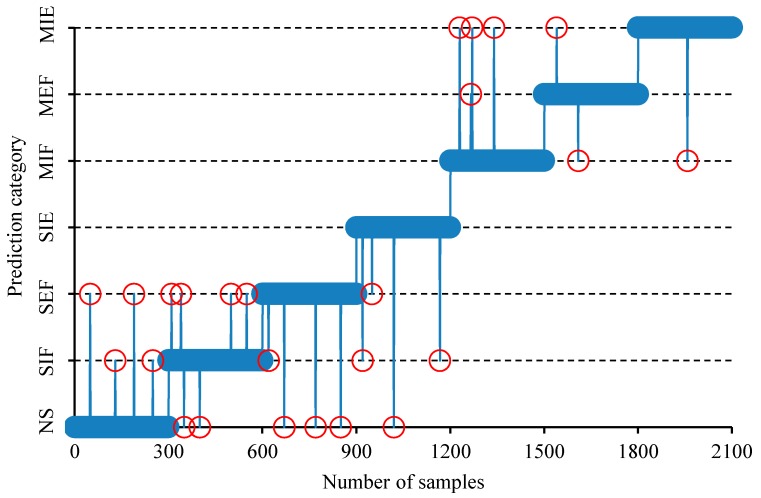
The detailed analysis of diagnostic result.

In order to further reflect the diagnostic effect of HSO–VSAE, a detailed analysis is carried out on the last test. In this test, there are 400 data files for each fault, and the total number of data files is 2800. The specific classification results are shown in Figure 11. It can be seen that there are four data files of NS that are misclassified, six data files of SIF are misclassified, four data files of SEF that are misclassified, four data files of SIE that are misclassified, four data files of MIF that are misclassified, two data files of MEF that are misclassified, and one data file of MIE that is misclassified. The overall accuracy rate reached 98.8%, indicating that the proposed HSO–VSAE method has a very satisfactory diagnostic effect on the valve clearance fault.

#### 5.4.2. Comparison with Baselines

To verify the fault diagnosis performance of the proposed HSO–VSAE method, comparisons with various existing diagnostic methods were carried out. In the KPCA+SVM method, the data were first reduced from 3600 to 60 dimensions by kernel principal components analysis (KPCA) with radial basis function (RBF) kernel, and then support vector machine (SVM) was used for fault classification. In the EEMD + KPCA + SVM method, the data were first adaptively decomposed into 11 intrinsic mode functions (IMFs) and a residual term by ensemble empirical mode decomposition (EEMD), then the dimensions of the 11 IMFs were reduced to 60 by KPCA. Finally, SVM was used for fault classification. In the multi-layer perceptron (MLP) method, the angular domain signals were taken as inputs, and the number of nodes in each layer was 3600-60-7 after HSO parameter optimization. In the SAE method, dropout layers and BN layers were added, and the number of nodes in each layer was 3600-500-250-60-7 after HSO parameter optimization. In the proposed HSO–VSAE method, five hyper-parameters were set by HSO algorithm, and the iteration epochs of AE1, AE2, and VAE in pre-training were 200, 100, 50 respectively, while the iteration epochs of fine-tuning was 20. For each percentage of the training set, 20 repeated tests were performed, and the statistical average accuracy and standard deviations are shown in Table 5. It can be seen that the proposed HSO–VSAE method is superior to the other algorithms in terms of diagnostic accuracy at each training data file ratio.

In order to study the diagnostic advantages of the proposed HSO–VSAE method under unknown operating conditions, comparisons of HSO-SAE and HSO–VSAE were carried out under different operating conditions. In the control experiment, the operating conditions in the test set were different from the operating conditions in the training set. The code of each operating condition is shown in Table 3, and the comparison results are shown in Table 6. It can be seen from Table 6 that the proposed HSO–VSAE method can cope well with the problem of variable operating conditions. The model can still show excellent fault diagnosis performance, even if the operating conditions in the test set have never appeared in the training set, because of the existence of the VAE part in the HSO–VSAE.

## 6. Conclusions

This work presents an effective diesel engine valve train fault approach based on the HSO–VSAE method, which extracts features hierarchically from the angular domain signals and can adaptively adjust the network structure [30]. In the proposed scheme, two AEs and a VAE are pre-trained to learn feature representations separately before they are stacked into VSAE. Then, the fine-tuning is performed to get a better model initialization. The advantages of the VSAE in extracting deep features directly from the angular domain signal are combined with the dropout technique, the BN technique, and the HSO algorithms, which make it possible to show excellent performance in the field of feature extraction. The visualization technique shows that the classification ability is getting better after each hidden layer of the proposed model. The experimental results prove that the proposed method can still perform well even without the help of signal processing based on expert knowledge, and outperform the KPCA + SVM, EEMD + KPCA + SVM, BP, and SAE methods in the diagnosis of valve clearance faults.

## Figures and Tables

**Table 1:** The key parameters of the diesel engine.

Item	Value
Shape	V-shaped 60°
Number of cylinders	12
Compression ratio	15:1
Rating speed	2100 r/min
Rating power	485 kW
Firing sequence	B1-A1-B5-A5-B3-A3-B6-A6-B1-A2-B4-A4

**Table 2:** The detailed fault valve train clearance setting.

Fault Category	Intake Valve Clearance (mm)	Exhaust Valve Clearance (mm)	Number of Data Files
NS	0.3	0.5	960
SIF	0.9	0.5	960
SEF	0.3	1.1	960
SIE	0.9	1.1	960
MIF	0.6	0.5	960
MEF	0.3	0.8	960
MIE	0.6	0.8	960

**Table 3:** The detailed operating conditions in the experiment.

Operating Condition	Speed (rpm)	Torque (N·m)
1	1500	700
2	1500	1000
3	1500	1300
4	1800	700
5	1800	1000
6	1800	1300
7	1800	1600
8	2100	700
9	2100	1000
10	2100	1300
11	2100	1600
12	2100	2200

**Table 4:** The optimized hyper-parameters in the harmony memory.

Hyper-Parameter Combination	*Dropout1*	*Dropout2*	*m*	*l*	*k*	Accuracy (%)
1	0.28	0.36	580	220	62	98.8
2	0.34	0.42	660	380	84	98.6
3	0.32	0.38	660	260	90	98.7
4	0.36	0.44	720	320	72	98.8
5	0.26	0.42	640	360	58	98.7
6	0.30	0.32	560	340	60	98.6
7	0.22	0.38	520	280	74	98.6

**Table 5:** Comparison of different diagnostic methods.

Method	Average Accuracy ± Standard Deviation (%)			
	20% Training Data Files	40% Training Data Files	60% Training Data Files	80% Training Data Files
KPCA + SVM	85.29 ± 0.74	86.78 ± 0.71	88.43 ± 0.73	88.35 ± 0.77
EEMD + KPCA + SVM	92.13 ± 0.85	92.57 ± 0.82	93.16 ± 0.74	92.62 ± 0.69
MLP	88.43 ± 0.93	89.64 ± 0.87	90.65 ± 0.72	90.88 ± 0.70
SAE	93.20 ± 0.69	93.65 ± 0.62	94.10 ± 0.58	93.97 ± 0.53
Proposed method	97.23 ± 0.45	98.16 ± 0.37	98.85 ± 0.26	98.79 ± 0.31

**Table 6:** Comparison under variable operating conditions.

Method	Operating Condition		Accuracy (%)
	Training Set	Test Set	
HSO-SAE	4~12	1~3	93.4
HSO-SAE	1~3, 8~12	4~7	93.8
HSO-SAE	1~7	8~12	93.2
HSO–VSAE	4~12	1~3	98.4
HSO–VSAE	1~3, 8~12	4~7	98.6
HSO–VSAE	1~7	8~12	98.3
